# Clinical characteristics of acute liver failure associated with hepatitis A infection in children in Mogadishu, Somalia: a hospital-based retrospective study

**DOI:** 10.1186/s12879-021-06594-7

**Published:** 2021-08-30

**Authors:** Esra Keles, Mohamed A. Hassan-Kadle, Marian Muse Osman, Hasan Huseyin Eker, Zeynep Abusoglu, Kursad Nuri Baydili, Aamir Muse Osman

**Affiliations:** 1 Department of Gynecologic Oncology, Zeynep Kamil Training and Research Hospital, University of Health Sciences Turkey, Istanbul, Turkey; 2 College of Medicine and Health Science, Abrar University, Mogadishu, Somalia; 3 SomGastro Clinic, Center For Liver Disease, Mogadishu, Somalia; 4 Department of Public Health, Mogadishu Somalia-Turkey Recep Tayyip Erdoğan Training and Research Hospital, University of Health Sciences Turkey, Mogadishu, Somalia; 5 Department of Public Health, Hamidiye Faculty of Medicine, University of Health Sciences Turkey, Istanbul, Turkey; 6 Department of Pediatrics, Mersin City Training and Research Hospital, Mersin, Turkey; 7 Department of Biostatistics, Hamidiye Faculty of Medicine, University of Health Sciences Turkey, Istanbul, Turkey; 8 Department of Parasitology, Abrar Research and Training Center, Abrar University, Mogadishu, Somalia

**Keywords:** Acute liver failure, Children, Hepatitis A, Somalia

## Abstract

**Background:**

Hepatitis A is one of the most common infectious causes of acute hepatitis, and currently, a neglected global public health problem necessitating an urgent response in Somalia. Hepatitis A infection and its rare complication of acute liver failure in children are largely based on very limited data. The aim of the study was therefore to investigate the Hepatitis A infection and its rare complication of acute liver failure in children in Somalia.

**Methods:**

This retrospective study was conducted on children aged 0–18 years who were admitted to the pediatric departments of the Somalia Mogadishu-Turkey Training and Research Hospital, Somali, from June 2019 and December 2019. Patients who were tested for hepatitis A infection during the study period and had complete data were included. Children with chronic disease, primary or secondary immunodeficiency, blood transfusion history, and missing data were excluded. Abstracted data including patients' demographics, clinical presentation, laboratory results, ultrasonographic findings, length of hospital stay, clinical course and outcome were retrieved from the hospital database system.

**Results:**

Of the 13,047 children, 219 were analyzed. Of the 219 Hepatitis A cases, 25 (11%) were diagnosed with pediatric acute liver failure (PALF). The mean age of children with Hepatitis A was 6.7 years. The majority of cases were reported in the 5–9 (39.7%) year age range. Hepatic encephalopathy, length of hospital stay, levels of albumin, and values of PT, aPPT, and INR were significantly higher in children with acute live failure. The presence of cholecystitis and cholecystitis with ascites in the sonographic evaluation were poor prognostic markers for acute liver failure.

**Conclusions:**

This study revealed hepatitis A virus infection and its related acute liver failure among hospitalized children in Somalia of which 11% had PALF. Hence, the introduction of Hepatitis A vaccination, which is the main public health tool, into the national immunization program, the improvement of hygiene conditions, raising awareness of the disease, and increasing health literacy are necessary to prevent the consequence of the Hepatitis A virus in children.

## Background

Hepatitis A is one of the most common infectious causes of acute hepatitis, but is currently, a neglected global public health concern necessitating an urgent response. It is a self-limiting, vaccine-preventable, non-chronic disease but rarely leads to acute liver failure [1]. State departments of health reported that the Hepatitis A outbreak resulted in 39,239 new cases and 372 deaths between July 2016 and July 2021 [2]. The causative pathogen is a Hepatitis A virus transmitted mainly through the fecal–oral route either by consumption of contaminated food and water or direct contact with an infected individual [3]. In Low and Middle-Income Countries (LMICs), this foodborne or waterborne disease is closely related to poor personal or environmental hygiene conditions and inadequate sanitation systems [3, 4].

Infection with HAV causes an immune response which is assessed by measurement of specific antibodies: immunoglobulin class M (IgM) anti-HAV antibodies and immunoglobin class G (IgG) anti-HAV antibodies and detected during acute illness when the serum aminotransferase levels are elevated and there is fecal shedding of the virus. Anti-HAV IgM antibodies can be detected after acute infection and antibodies often disappear up to 6 months, while Anti-HAV IgG antibodies present within 2 to 3 months after the onset of the disease and protect against infections by providing life-long immunity [5, 6]. Most studies on seroprevalence data of hepatitis A virus are usually reported as anti-HAV IgG, rather than anti-HAV IgM seroprevalence data [3].

The hepatitis A infection includes a wide range of clinical manifestations from asymptomatic disease to symptomatic diseases presents as jaundice, fever, malaise, anorexia, nausea, and abdominal discomfort. While a majority of Hepatitis A cases resolve spontaneously or with supportive treatments, about 0.1–0.4% of pediatric cases are complicated by acute liver failure, with a reported mortality rate in cases without access to liver transplantation is over 80% [7]. Pediatric acute liver failure (PALF) is an extremely rare complication of Hepatitis A, one of the major causes, may require liver transplantation or result in death due to low transplant-free survival, which is of great concern due to its severe consequences [8]. Our knowledge of Hepatitis A infection and related complications, particularly acute liver failure, in children is largely based on very limited data [9, 10]. The aim of the research was therefore to investigate the impact of pediatric Hepatitis A infection and its consequences, especially acute liver failure, in Somalia, a low-income country. In addition, this study provides the first comprehensive assessment of pediatric acute liver failure due to Hepatitis A infection after the civil war in Somalia, 1992.

Hepatitis A virus infection depends on global socio-economic conditions. In high-income countries, the prevalence of HAV is very low (< 50% of the population), and in middle-income countries is at a medium or low level, while in low-income countries is high (> 90% of the population), where almost all the children were infected with HAV (> 90%), particularly by the ages of 10 [1, 3, 11]. The current prevalence of hepatitis A virus in Somalia was 90% while the children population prevalence rates were 96% [12].

Somalia is a highly endemic country for hepatitis A infection, only a few studies have been carried out on the hepatitis A infection and the factors affecting the clinical course of this disease in children over the past decades, which needs robust data in this issue. Therefore, in the presented paper, we aimed to examine the demographic characteristics, clinical and laboratory findings, and clinical course of pediatric age groups who developed acute liver failure due to hepatitis A infection.

## Materials and methods

This retrospective study was conducted on children aged 0–18 years who were admitted to the pediatric outpatient clinic and pediatric emergency departments of the Somalia Mogadishu-Turkey Training and Research Hospital, Somali, from June 2019 and December 2019. Our institution is the largest tertiary referral health facility in the region with a 200-bed capacity and serving approximately 327 thousand patients annually.

Data were retrieved from the hospital database system for the records of patients < 18 years of age who were tested for Hepatitis A infection at our institution. Patients who were tested for hepatitis A infection during the study period and had complete data were included in the study. Children with chronic disease, primary or secondary immunodeficiency, blood transfusion history, and missing data were excluded from the study. Clinicians were unable to test most children with mild Hepatitis A infection who could be treated on an outpatient basis due to the cost of testing in this low-resource setting. Hepatitis A testing has often been performed for children who required hospitalization due to severe illness. Abstracted data included patients' demographics (age, gender), clinical presentation (jaundice, fever, vomiting, abdominal pain, asymptomatic), complete blood count (hemoglobin, platelet), routine liver function tests (Alanine transaminase, Aspartate transaminase, Alkaline phosphatase, Gamma-glutamyltransferase, Total and direct bilirubin), ultrasonographic findings (hepatomegaly, presence of ascites, cholecystitis, normal), blood coagulation system parameters (Standardization of Prothrombin Time/International Normalized Ratio [PT/INR], activated partial thromboplastin time [APTT]), albumin level, length of hospital stay, clinical course and outcome (the presence or absence of hepatic encephalopathy, chronic hepatitis, death, recovery). Pediatric acute liver failure was defined according to the pediatric acute liver failure of the study group (PALFSG) criteria [13]. The PALFSG has defined acute liver failure as (1) the presence of severe hepatic dysfunction occurring within 8 weeks of the onset of illness, (2) child aged 0–18 years with no known chronic liver disease (3) biochemical evidence of acute liver injury (4) liver-based coagulopathy defined as PT ≥ 15 s or INR ≥ 1.5 not corrected by vitamin K in the patients with hepatic encephalopathy or PT ≥ 20 s or INR ≥ 2.0 in patients with or without hepatic encephalopathy [8, 13]. Database management complies with legislation on privacy and this research is in accordance with the ethical principals of the Declaration of Helsinki and approval for this retrospective research on de-identified hepatitis A patients was obtained from the Somalia Mogadishu–Turkey Recep Tayyip Erdogan Training and Research Hospital Ethics Committee (26.12.2019-MSTH/2903). Informed consent was waived due to the retrospective study design by the same ethics committee that approved this study (Somalia Mogadishu–Turkey Recep Tayyip Erdogan Training and Research Hospital).

All data analysis was performed using IBM SPSS Statistics for Windows, Version 25 (IBM Corp. Armonk, N.Y., USA). Continuous variables were expressed as mean and standard deviation (mean ± SD), while categorical variables were as percentages. The relationship between categorical variables was determined using Fisher exact test and the Chi-squared test. A p value of ≤ 0.05 was considered statically significant.

## Results

**Fig. 1:**
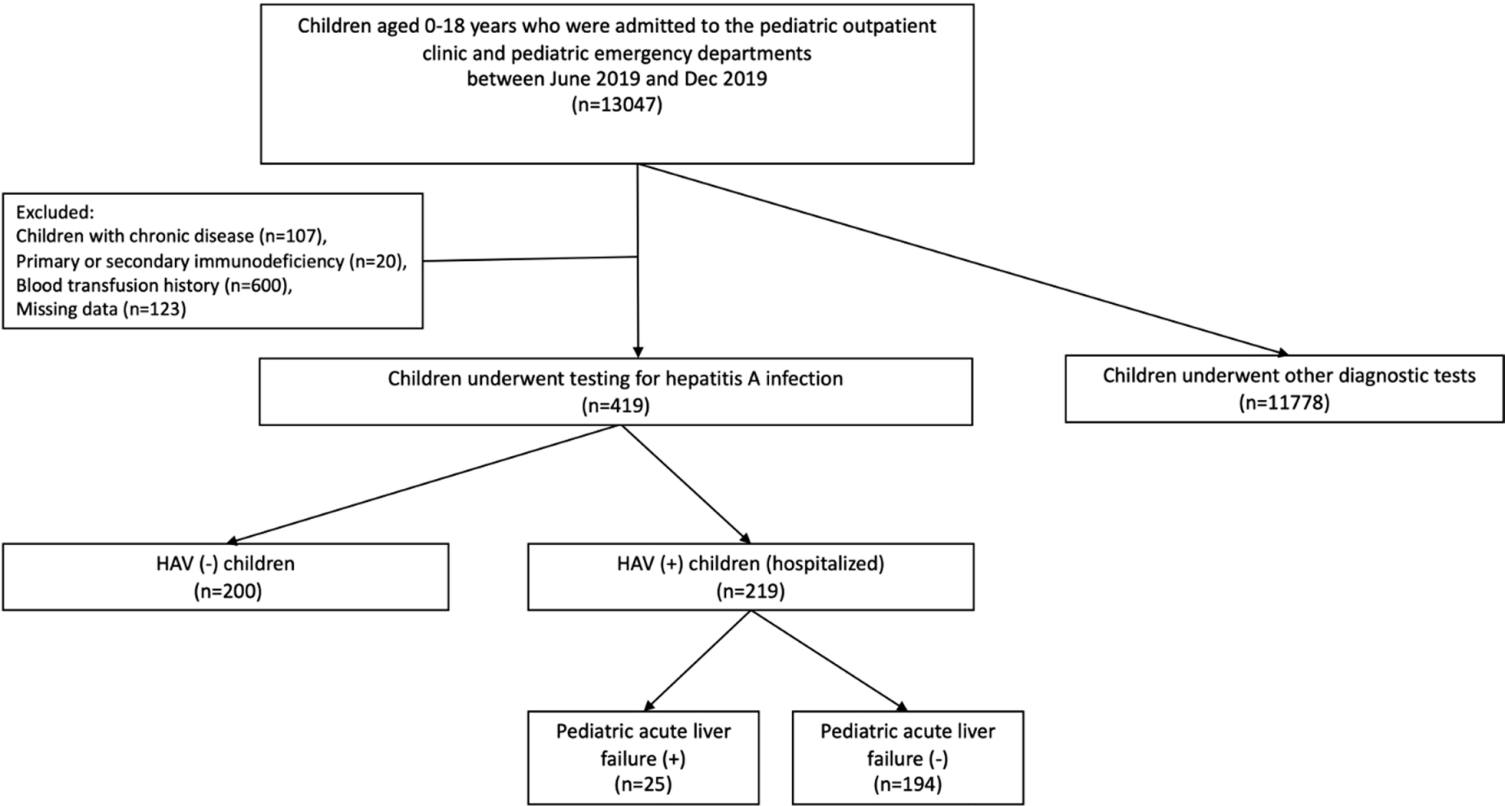
The flow diagram of the study

Between June 2019 and December 2019, of the 13,047 patients admitted to the pediatrics department, 419 were tested for Hepatitis A IgM and IgG tests. Among 419 patients, 219 were diagnosed with hepatitis A infection and necessitating a further comprehensive examination by hospitalization. Of the 219 Hepatitis A positive cases enrolled in this retrospective study, 25 (11%) were diagnosed with pediatric acute liver failure (PALF), while 194 (89%) were not diagnosed with PALF (Fig. 1).

**Fig. 2:**
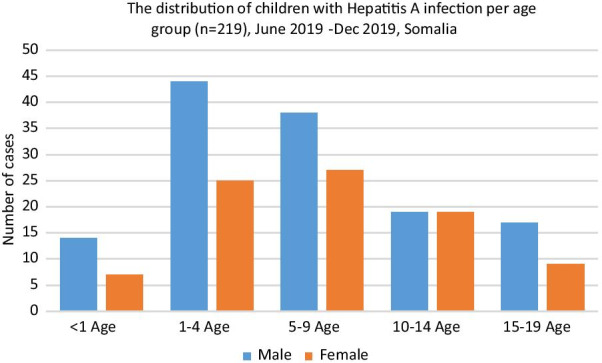
The distribution of children with Hepatitis A infection per age group (n=219), June 2019-Dec 2019, Somalia

The age distribution of children with Hepatitis A infection was as follows: < 1 year, 9.6% (n = 21); 1 to 4 years, 31.5% (n = 69); 5 to 9 years, 39.7% (n = 65); 10 to 14 years, 17.4% (n = 38) and 14 to 19 years 11.9% (n = 26) (Fig. 2). In the Hepatitis A infection positive group of 219 children (132 male, 87 female; mean age 6.7 years) were examined in two groups; one which was an acute liver failure was observed (n = 25; 16 male, 9 female; mean age = 6.7 years) and another which it was not (n = 194; 116 male, 78 female; mean age = 7.1 years) (p = 0.851).

In the comparison of HAV positive patients according to clinical findings including jaundice, fever, vomiting and asymptomatic at hospital admission; It was found as 6 (35%), 0 (0.0%), 3 (10%), 16 (12%) in the PALF positive group, while in the non-PALF group it was 11 (65%), 33 (100%), 28 (90%), 122 (88%) (p = 0.004).

Sonographic imaging information was available for 84 patients. Detailed abdominal ultrasonography revealed cholecystitis in 3 (21%) patients, hepatomegaly and ascites in 4 (80%) patients, cholecystitis and ascites in 1 (8%) patient in the acute liver failure group; while this was in 11 (79%), 1 (20%), 11 (92%) patients in the non-hepatic failure group, respectively (p < 0.001).

Children with acute liver failure significantly had more prolonged PT and aPPT, and higher INR values in coagulation assays; and had higher levels of albumin in biochemical tests than the group without liver failure (for all, p ≤ 0.05). In terms of other laboratory parameters, there were no significant differences between the two groups.

In relation to the clinical outcomes of the HAV-positive children, the most commonly identified clinical result was the recovery with supportive treatment in both groups (< 0.001). Hepatic encephalopathy was observed in individuals with hepatitis A disease (12/219; 15.4%), in which PALF positive group (5/25;40%) was significantly higher compared to the non-PALF group (7/194; 4%) (p =  < 0,001). The length of stay in the hospital or intensive care unit was significantly higher in children with acute liver failure (p = 0.001). The clinical presentation at hospital admission, sonographic findings, laboratory parameters, clinical outcome, and length of stay in the hospital or intensive care unit of patients diagnosed with and without pediatric liver failure due to hepatitis A infection are presented in Table 1.

**Table 1:** Characteristics of hepatitis A patients by pediatric acute liver failure (PALF−/+) (n = 219), June 2019–Dec 2019, Somalia

Characteristics	PALF (−)	PALF (+)	p value
	(n = 194)	(n = 25)	
Age (mean ± SD) (years)	6.7 ± 4.8	7.1 ± 6.8	0.802
Gender, N (%)			
Male	116 (88)	16 (12)	
Female	78 (90)	9 (10)	0.851
Clinical presentation, N (%)			
Jaunce	11 (65)	6 (35)	
Fever	33 (100)	0 (0)	0.004*
Vomiting	28 (90)	3 (10)	
Asymptomatic	122 (88)	16 (12)	
Sonographic findings, N (%) (n = 84)			
Normal	33 (94)	2 (6)	
Hepatomegaly	3 (50)	3 (50)	
Ascites	7 (58)	5 (42)	< 0.001*
Cholecystitis	11 (79)	3 (21)	
Hepatomagly + ascites	1 (20)	4 (80)	
Cholecystitis + ascites	11 (92)	1 (8)	
Laboratory results (mean ± SD)			
Platelet (× 10^9^/L)	315 ± 186	347 ± 500	0.753
Total bilirubin (μmol/L)	22 ± 118	247 ± 572	0.062
Direct bilirubin (μmol/L)	14 ± 96	53 ± 84	0.058
Albumin (g/L)	5 ± 11	11 ± 14	0.038*
ALT (U/L)	517 ± 714	1420 ± 2118	0.045*
AST (U/L)	612 ± 936	2760 ± 5630	0.069
APTT (s)	6 ± 12	155 ± 233	0.004*
GGT (U/L)	57 ± 137	54 ± 51	0.84
INR	0,3 ± 0,5	147 ± 301	0.022*
PT (s)	3,7 ± 10	149 ± 286	0.018*
Clinical results, N (%)			
Recovery	174 (92)	15 (8)	< 0.001*
Relaps	13(72)	5 (28)	
Hepatic encephalopathy	7 (58)	5 (42)	
Length of hospital stay (days) (mean ± SD)	1.61 ± 3.5	5.88 ± 5.6	0.001*

Normal values: ALT (5–45 U/L), AST (5–40 U/L), alkaline phosphatase (38–155 U/L), albumin (2.5–6.0 g/dL), total serum bilirubin (0.1–1.3 mg/dL) and serum direct bilirubin (0.0–8.4 mg/dL)

*ALT* alanine aminotransferase; *AST* aspartate aminotransferase; *PT* prothrombin time; *GGT* gamma glutamyl transpeptidase; *INR* International normalized ratio; *PT* prothrombin time

*p < 0.05

## Discussion

Hepatitis A virus remains a worldwide public health issue and is endemic in many low-middle income countries. The overall prevalence of hepatitis A virus in Somalia was 90% (12), which is to be a high-level prevalence. Compared to the previous study of the country in 1992 in children were 59% and 96% in two institutions at Mogadishu city [14]. While comparing to countries the prevalence of hepatitis A is similar to those reported from Eastern Mediterranean Region (EMRO) such as Palestine 93.7% in school children [15] and Syria 95% in the 11–15 years old children [16] and sub-Saharan region such as South Africa 90% [3]. But markedly different from Kenya 44.5% and Nigeria 55.2% in the pediatric population [17, 18]. Previously studies proposed that hepatitis A was highly prevalent in the region. However, these calculations were only based on the limited research after the Somalia civil war era, from 1992 and 2000, but our findings on the frequency of children aged ≤ 18 years with Hepatitis A much lower, would thus seem to be defensible and need to be validated with future research.

Recent studies showed that hepatitis A virus is the most common cause of PALF than other viral infections or diseases in children in low-middle income countries [19–21]. In our study showed that PALF was diagnosed in 11% (25/219) of the study population. It is worth mentioning that supporting preventive measures to tackle the burden of disease, such as initiating vaccination as the main public health tool, improving safe drinking water, the sanitary system, hygiene conditions, food safety, increasing health literacy, and raising disease awareness are crucial interventions for countries aiming to eliminate this preventable disease and its complications.

When we compare the distribution of hepatitis A infection according to the age group, our results concur well with the surveillance data of which reported to have the highest hepatitis A incidence in children aged 5–14 [22], and but differs with respect to those reported by Lankarani's study which showed the majority of the study group constituted of 15–19 age group [23]. In the present study, HAV infection is more common in males than females in all age groups. In the population-based study of Wu et al., higher rates of Hepatitis A infection were shown for males than for females except in the 5–19 age group [24], while Khalil et al. [25] and Faleh et al. [26] showed higher seropositivity for males in their studies. The difference in the HAV seropositivity rates between males and females may be linked to the risk activities in society [24–26].

In the presented article, it has been demonstrated that the presence of cholecystitis and cholecystitis with ascites in pediatric Hepatitis A cases were poor prognostic markers confirming previous findings in the literature [27]. The presence of these ultrasonographic findings may serve as predictors of unfavorable prognosis in pediatric acute liver failure patients, which could help clinicians to tailor the patient management in low-sources settings.

Hepatitis A infection is endemic in poor sanitary and hygienic conditions. The high prevalence of Hepatitis A infection could be attributed to overcrowding, lack of access to a safe primary water supply system, absence of a routine population-based vaccination schedule against Hepatitis A infection. Although death rates of Hepatitis A infection seem to be low, require long-term hospitalization of patients due to the complication of acute liver failure, which causes loss of workforce, constitutes a socio-economic burden on individuals and healthcare systems, and leads to mortality in settings where referral pediatric liver transplantation centers are not available [28]. World health organization recommends the introduction of the vaccination program on a large-scale in high-to-intermediate Hepatitis A infection endemic regions, with additional support programs such as improved sanitary systems, as well as increased health literacy resulting in an increment in the personal hygiene practices [21].

We are aware that there may be several limitations that need to be acknowledged. The first is the retrospective design of the study. Despite being the largest hospital in the region and having a significant annual number of admissions, clinicians were unable to perform serological tests from all patients due to the cost of the assays and access to the available complete data of those tested constitute the reasons for the small sample size of the study, which is another limitation. Notwithstanding these limitations, the study has some strengths that represent the first comprehensive examination of the Hepatitis A infection and its rare complication of acute liver failure in the pediatric age group through a public health perspective after the civil war in Somalia, 1991. Additionally, this paper provides valuable insights into the growing body of literature on Hepatitis A infection and its clinical outcomes and guides to both practitioners and policy-makers. We are confident that our research will serve as a base for future studies and for public health actions on hepatitis infections and its consequences to establish a greater degree of awareness.

## Conclusions

This study highlights the significance of Hepatitis A infection and HAV-related acute liver failure among hospitalized children in Somalia. Implementing urgent public health strategies is required to minimalizing the impact of Hepatitis A infection in Somalia after the civil war. Policymakers should act fast and formulate scientific work into effective health policies in order to eliminate this curable disease. Hence, the introduction of Hepatitis A vaccination, which is the primary prevention method from HAV infection, into the national immunization program, the improvement of hygiene conditions, raising awareness of the disease, and increasing literacy of the community is necessary to prevent the Hepatitis A infection and its consequences.

## Data Availability

The dataset used and/or analyzed in the study are available from the corresponding author on reasonable request.

## Abbreviations

HAV: Hepatitis A virus
IgG: Immunoglobin class G antibodies
IgM: Immunoglobin class M antibodies
EMRO: Eastern Mediterranean Region
LMICs: Low and Middle-Income Countries
PALF: Pediatric acute liver failure
PALFSG: Pediatric acute liver failure of the study group

## References

[1] Franco E, Meleleo C, Serino L, Sorbara D, Zaratti L (2012). Hepatitis A: epidemiology and prevention in developing countries. World J Hepatol.

[2] Centers for Disease Control and Prevention. Widespread person-to-person outbreaks of hepatitis A across the United States. In: Centers for Disease Control and Prevention. https://www.cdc.gov/hepatitis/outbreaks/2017March-HepatitisA.htm.

[3] Patterson J, Abdullahi L, Hussey GD, Muloiwa R, Kagina BM (2019). A systematic review of the epidemiology of hepatitis A in Africa. BMC Infect Dis.

[4] Khan AI, Salimuzzaman M, Islam MT, Afrad MH, Shirin T, Jony MH (2020). Nationwide hospital-based seroprevalence of hepatitis A and hepatitis E virus in Bangladesh. Ann Glob Health.

[5] Samaddar A, Taklikar S, Kale P, Kumar CA, Baveja S (2019). Infectious hepatitis: A 3-year retrospective study at a tertiary care hospital in India. Indian J Med Microbiol.

[6] Rodríguez Lay Lde L, Larralde Díaz O, Martínez Casanueva R, Gutiérrez Moreno A (2003). Anti-hepatitis A virus immunoglobulin M antibodies in urine samples for rapid diagnosis of outbreaks. Clin Diagn Lab Immunol.

[7] Diniz-Santos DR, Melo MC, Melo RF, Silva LR (2004). Acute liver failure complicating viral hepatitis A. Braz J Infect Dis.

[8] Ng VL, Li R, Loomes KM, Leonis MA, Rudnick DA, Belle SH (2016). Pediatric Acute Liver Failure Study Group (PALFSG). Outcomes of children with and without hepatic encephalopathy from the pediatric acute liver failure study group. J Pediatr Gastroenterol Nutr.

[9] Oh SH, Kim KM, Kim DY, Kim Y, Song SM, Lee YJ (2014). Improved outcomes in liver transplantation in children with acute liver failure. J Pediatr Gastroenterol Nutr.

[10] Devarbhavi H, Singh R, Adarsh CK, Sheth K, Kiran R, Patil M (2014). Factors that predict mortality in children with Wilson disease associated acute liver failure and comparison of Wilson disease specific prognostic indices. J Gastroenterol Hepatol.

[11] Havelaar AH, Kirk MD, Torgerson PR, Gibb HJ, Hald T, Lake RJ (2015). World Health Organization Foodborne Disease Burden Epidemiology Reference Group. World Health Organization global estimates and regional comparisons of the burden of foodborne disease in 2010. PLoS Med.

[12] Hassan-Kadle MA, Osman MS, Ogurtsov PP (2018). Epidemiology of viral hepatitis in Somalia: systematic review and meta-analysis study. World J Gastroenterol.

[13] Squires RH, Shneider BL, Bucuvalas J, Alonso E, Sokol RJ, Narkewicz MR (2006). Acute liver failure in children: the first 348 patients in the pediatric acute liver failure study group. J Pediatr.

[14] Bile K, Mohamud O, Aden C, Isse A, Norder H, Nilsson L (1992). The risk for hepatitis A, B, and C at two institutions for children in Somalia with different socioeconomic conditions. Am J Trop Med Hyg.

[15] Yassin K, Awad R, Tebi A, Queder A, Lasser U (2001). The epidemiology of hepatitis A infection in Palestine: a universal vaccination programme is not yet needed. Epidemiol Infect.

[16] Antaki N, Kebbewar MK (2000). Hepatitis A seroprevalence rate in Syria. Trop Doct.

[17] Wasunna A, Murila F, Obimbo MM, Rama MJ, Musembi H (2016). Hepatitis A antibody seroprevalence in a selected Kenyan pediatric population. Open J Pediatr.

[18] Ikobah JM, Okpara HC, Ekanem EE, Udo JJ (2015). Seroprevalence and predictors of hepatitis A infection in Nigerian children. Pan Afr Med J.

[19] Pandit A, Mathew LG, Bavdekar A, Mehta S, Ramakrishnan G, Datta S (2015). Hepatotropic viruses as etiological agents of acute liver failure and related-outcomes among children in India: a retrospective hospital-based study. BMC Res Notes.

[20] Alam S, Khanna R, Sood V, Lal BB, Rawat D (2017). Profile and outcome of first 109 cases of paediatric acute liver failure at a specialized paediatric liver unit in India. Liver Int.

[21] Sood V, Lal BB, Gupta E, Khanna R, Siloliya MK, Alam S (2019). Hepatitis A virus-related pediatric liver disease burden and its significance in the Indian subcontinent. Indian Pediatr.

[22] Centers for Disease Control and Prevention: Hepatitis surveillance report no. 57. Atlanta Centers for Disease Control and Prevention. https://stacks.cdc.gov/view/cdc/23152 (2000). Accessed 8 Mar 2021.

[23] Lankarani KB, Mahmoodi M, Honarvar B, Nematollahi P, Zamiri N, Ghaffarpasand F (2014). Determinants of poor outcome in patients with hepatitis A infection: a four-year retrospective study in Shiraz Southern Iran. Arch Virol.

[24] Wu J, Zou S, Giulivi A (2001). Current hepatitis A status in Canada. Can J Infect Dis.

[25] Khalil M, Al-Mazrou Y, Al-Jeffri M, Al-Howasi M (1998). Childhood epidemiology of hepatitis A virus in Riyadh Saudi Arabia. Ann Saudi Med.

[26] Al Faleh F, Al Shehri S, Al Ansari S, Al Jeffri M, Al Mazrou Y, Shaffi A (2008). Changing patterns of hepatitis A prevalence within the Saudi population over the last 18 years. World J Gastroenterol.

[27] Ewadh ZG, Abd BA, Al-Khafaji HF, Abd BA (2020). Pediatric hepatitis A from ultrasonographic, immunologic, and other points of view. Ann Trop Med Health.

[28] Jacobsen KH (2009). The global prevalence of hepatitis A virus infection and susceptibility: a systematic review.

